# ‘The Future is Probably Now’: Understanding of illness, uncertainty and end‐of‐life discussions in older adults with heart failure and family caregivers

**DOI:** 10.1111/hex.12980

**Published:** 2019-09-27

**Authors:** Jennifer Im, Susanna Mak, Ross Upshur, Leah Steinberg, Kerry Kuluski

**Affiliations:** ^1^ Institute of Health Policy, Management, and Evaluation University of Toronto Toronto Ontario Canada; ^2^ Division of Cardiology Mount Sinai Hospital Sinai Health System Toronto Ontario Canada; ^3^ Institute of Medical Sciences University of Toronto Toronto Ontario Canada; ^4^ Lunenfeld‐Tanenbaum Research Institute Sinai Health System Toronto Ontario Canada; ^5^ Dalla Lana School of Public Health University of Toronto Toronto Ontario Canada; ^6^ Department of Family & Community Medicine University of Toronto Toronto Ontario Canada; ^7^ Temmy Latner Centre for Palliative Care Mount Sinai Hospital Sinai Health System Toronto Ontario Canada; ^8^ Institute for Better Health Trillium Health Partners Mississauga Ontario Canada

**Keywords:** advanced illness, caregivers, end‐of‐life communication, end‐of‐life discussions, heart failure, older adults, qualitative research, uncertainty, understanding of illness

## Abstract

**Background:**

Earlier end‐of‐life communication is critical for people with heart failure given the uncertainty and high‐risk of mortality in illness. Despite this, end‐of‐life communication is uncommon in heart failure. Left unaddressed, lack of end‐of‐life discussions can lead to discordant care at the end of life.

**Objective:**

This study explores patients' and caregivers’ understanding of illness, experiences of uncertainty, and perceptions of end‐of‐life discussions in advanced illness.

**Design:**

Interpretive descriptive qualitative study of older adults with heart failure and family caregivers. Fourteen semi‐structured interviews were conducted with 19 participants in Ontario, Canada. Interviews were transcribed verbatim and content analysis was used to analyse the data.

**Main results:**

Understanding of illness was shaped by participants’ illness‐related experiences (e.g. symptoms, hospitalizations and self‐care routines) and the ability to adapt to challenges of illness. Participants were knowledgeable of heart failure management, and yet, were limited in their understanding of the consequences of illness. Participants adapted to the challenges of illness which appeared to influence their perception of overall health. Uncertainty reflected participants’ inability to connect manifestations of heart failure as part of the progression of illness towards the end of life. Most participants had not engaged in prior end‐of‐life discussions.

**Conclusion:**

Detailed knowledge of heart failure management does not necessarily translate to an understanding of the consequences of illness. The ability to adapt to illness‐related challenges may delay older adults and family caregivers from engaging in end‐of‐life discussions. Future research is needed to examine the impact of addressing the consequences of illness in facilitating earlier end‐of‐life communication.

## 1 INTRODUCTION

Chronic heart failure is a progressive illness characterized by periodic exacerbations, uncertainty in the illness trajectory, and high symptom burden that can lead to repeated interruptions in quality of life.^1^ Heart failure affects over 26 million people globally.^2^ Although survival has improved over the last decade, over 50% of patients still die within 5 years of being diagnosed.^3^ Despite this evidence, it has been reported that <40% of people with heart failure are aware of their diagnosis.^4^ Older adults experience additional challenges, such as with multimorbidity and frailty, that increase the complexity of their illness. The increased complexity further compounds uncertainty in illness.^5^, ^6^ The complexity of managing multiple illnesses and its unpredictable course has been found to contribute to a poor understanding of illness. Multiple reviews have found that most patients and caregivers do not receive prognostic information and have unmet information needs.^7^, ^8^, ^9^, ^10^ A poor understanding of illness can further lead to uncertain experiences for people with advanced illnesses such as heart failure.^11^

Clinical interactions that are designed to improve understanding of illness and to identify individual's goals of care are infrequently integrated into chronic disease management. End‐of‐life communication, which includes goals‐of‐care discussions, aims to create a shared understanding of an individual's values and preferences among patients, caregivers and clinicians.^12^ The integration of end‐of‐life communication early in the course of illness has been endorsed by multiple professional associations including the Canadian Cardiovascular Society, the American Heart Association, and the European Heart Association.^13^, ^14^, ^15^ Yet, end‐of‐life communication still occurs late in the course of illness, if at all, for people with advanced illness such as heart failure.^16^, ^17^

Integration of end‐of‐life communication into heart failure management is one approach to address the uncertainties of illness and improve understanding. It also has the potential to shift the delivery of care from being disease‐oriented to person‐centred, thereby providing support to individuals based on their identified goals and values.^18^ However, clinicians report lack of understanding of illness among patients and caregivers to be the most important barrier of end‐of‐life communication.^19^ To overcome these barriers in clinical practice, there is a need to better understand patient and caregiver perceptions of end‐of‐life discussions within the context of their understanding of illness. This study explores what patients and caregivers understand about their illness and how their understanding relates to their experience of uncertainty and end‐of‐life discussions in advanced illness.

## 2 METHODS

### 2.1 Study design and theoretical framework

This qualitative study was conducted following an interpretive description approach.^20^ Interpretive description is a pragmatic methodology that aims to generate knowledge that is useful and applicable in clinical practice.^21^ Interpretive qualitative research acknowledges the constructed and context‐dependent nature in which health‐related experiences form.^20^, ^21^ This study was further guided by the Reconceptualized Uncertainty in Illness Theory by Merle Mishel.^22^ Mishel defines uncertainty as the inability to attach meaning to illness‐related events (e.g. symptoms and hospitalizations) that provide the individual with information about their illness.^23^ One's ability to process illness‐related information depends on their prior experience of similar events and gained familiarity over time. It can also be influenced by their care team, which includes health‐care providers and informal caregivers (e.g. family members), as well as their own cognitive capacity to process illness‐related information. These antecedents accumulate to an individual's ability to understand illness‐related experiences. Uncertainty in illness develops when there are gaps in understanding of illness. For people with chronic illnesses, as experiences of uncertainty persist over time, the theory posits that prolonged uncertainty will push individuals to adjust to a life with uncertainty. The Reconceptualized Uncertainty in Illness Theory has been used in previous research of older adults with advanced illness.^11^

### 2.2 Setting and participants

Participants were recruited from an outpatient clinic in an academic health sciences centre in a metropolitan city in Ontario, Canada. A multidisciplinary team of clinicians with subspecialty training in heart failure management operates the specialized heart failure clinic. Purposeful sampling technique was used to recruit patients 65 years or older with a clinical diagnosis of advanced heart failure (New York Heart Association Class III/IV) and/or older adults over the age of 80 from the heart failure clinic (see Box 1). We specifically sought patients that meet these criteria as the challenges of illness are amplified in advanced stages of heart failure and as older adults often have multiple chronic conditions that challenge their understanding and management of illness. Patients who had already been referred to palliative care specialists were intentionally excluded. This decision was made as we purposely sought to sample patients and caregivers earlier in the illness trajectory, before the end‐of‐life, to explore perception of earlier end‐of‐life communication in advanced illness, and because current practices, in Ontario, reflect late referral to palliative care for patients with advanced illness.^24^ Family caregivers of patients, when available, were invited to participate in the study.

Box 1 **Inclusion criteria for participant recruitment**

- Patients aged 65 years and older with advanced heart failure (NYHA Class III/IV) or aged 80 years and older under care in the heart failure clinic
- Not waiting for a transplant
- Not under care by a specialist palliative care provider
- Have adequate stamina to complete an hour‐long interview
- Able to provide informed consent
- English speaking

To identify study candidates, the lead author (JI) met two cardiologists. Candidates were sent information letters prior to their appointment to notify them of the study. On the days of candidates’ appointments, at the end, the cardiologists sought permission from the patient, and when available their family caregiver, for a researcher to speak to them about the study. For interested candidates, JI explained the study purpose and asked for their participation in approximately an hour‐long interview. Participants were given the informed consent form and offered a choice to be interviewed in the clinic or in their homes. In total, 23 candidates were approached, of them, 19 participated, two patients and one caregiver declined, and one candidate could not be approached due to cognitive decline. After completing 14 interviews with 12 patients and seven family caregivers, saturation of key content was reached as determined by the lack of new data being generated on key phenomena of interest.

### 2.3 Data collection

The data collection process began by gathering information on participant demographics (e.g. age, sex, living arrangement and chronic conditions). Semi‐structured interviews with patients and family caregivers were conducted individually or as dyads depending on their preference. Interview guides were developed by JI with input from an interdisciplinary team (i.e. clinicians and researchers in cardiology, palliative care, family medicine and social work). Participants were asked open‐ended questions on three categories of interest: understanding of illness, goals of care, and end‐of‐life discussions (see Box 2). Adaptations were made to the guides as data collection and analysis progressed to refine questions and exhaust emerging concepts.

Box 2 **Semi‐structured interview guide**

1. I understand that you’ve been diagnosed with heart failure. When were you first diagnosed? How did you find out?
  Prompt: What issues have you had with your heart?
  Prompt: What do you understand about your health?
  Prompt: What have health‐care professionals told you about your heart?
2. What has your experience with heart failure [or other health issues] been like?
  Prompt: have you had to go to the emergency department or been hospitalized? What was it like?
3. What are some things you enjoy doing in your day‐to‐day life?
  Prompt: have you experienced any challenges/what are you unable to do due to your heart/health issues?
4. Can you tell me about some things that are important to you in your life?
5. Have you thought about the end‐of‐life before? In what ways?
6. Have you talked about end‐of‐life care before?
  Prompt: How did the conversation go?
7. Hypothetically, if you were to decline in the next few weeks, what would you say are important to you?
  Prompt: What would your preferences for the end‐of‐life be?
8. Have you discussed end‐of‐life care with your health‐care provider or family members?
  Prompt: Can you describe the process/conversation to me?
9. Why do you think you and your provider haven’t talked about the future or the end‐of‐life?
10. Do you think it’s important that your health‐care providers know what’s important to you?

Interviews were conducted by JI, who at the time was a Master's candidate in a health services research programme. JI had been mentored by the senior author (KK) for 3 years at the time. The senior author (KK) is an experienced qualitative researcher who leads a research programme on the experience of vulnerable populations and their caregivers. JI introduced herself as a Master's student who was conducting the study for her thesis. Half of the interviews were conducted at the clinic in a private room and the remainder of the interviews was conducted in participants’ homes. Before beginning the interviews, participants were reminded that the interviews would be audio‐recorded and transcribed verbatim by a medical transcriptionist. Participants were reminded that the transcripts would be checked for accuracy against the audio files at which point, any identifiable information would be redacted. Participants were also reminded that their participation was voluntary, that they could skip any questions, or end the interviews at any point. Data collection occurred between August 2017 and January 2018. No participants withdrew from the study. No prior relationship between the interviewer and any participants existed. The interviews lasted from 25 to 90 minutes and were completed in one session.

### 2.4 Data analysis

The initial analysis was performed inductively and simultaneously with data collection to exhaust the categories of interest using NVivo11 (QSR International). The in‐depth analysis of data began after all interviews had been conducted. Content analysis was used to categorize data according to the specific research questions under inquiry. Content analysis allows for inferences to be made from the data in its context to provide insights on a phenomenon, and it is an analytic technique that is flexible to both inductive and deductive analysis.^25^ Given the alignment between the research aim and the Reconceptualized Uncertainty in Illness Theory, a hybrid of conventional content analysis and a directed approach to content analysis was used. Conventional content analysis is typically used to describe and analyse data in the absence of a theoretical framework or when understanding of a phenomenon is limited, while directed approach to content analysis is used to extend conceptually a theoretical framework.^26^ The in‐depth analytical process began by deductively analysing the data into three broad categories: understanding of illness, uncertainty and end‐of‐life communication. This process involved reviewing the transcripts iteratively to code the data as they related to the identified categories. Within these broad categories, the data were analysed inductively and codes were generated to gain a deeper understanding of the phenomena within and across the categories. Regular meetings were held between the lead and senior author (JI and KK) to discuss findings as they developed, and check‐in meetings were held with all authors as analysis progressed. To verify the categories and interpretation of the data, the senior author (KK) reviewed three transcripts in depth, reviewed all codes and descriptions, the accompanying excerpts and interpretations, and posed questions to challenge the findings. This step was included to ensure that the interpretations were defensible and to increase the dependability of the findings.

### 2.5 Ethics

Prior to data collection, the study was approved by the Research Ethics Boards at Mount Sinai Hospital (July 12, 2017). All participants provided consent for their interview to be recorded, transcribed, and for the findings to be shared through presentations and publications.

## 3 RESULTS

### 3.1 Participant characteristics

Fourteen interviews were conducted with 19 participants (12 patients and 7 family caregivers). The mean age of patients was 82.5 years (SD = 6.4); the majority were male (58%), lived with their spouse (58%), and reported a mean of 5 chronic conditions (SD = 2.2) (see Tables 1 and 2). The mean age of caregivers was 67 years (SD = 13.7), and the majority were female (71%) (Table 3).

**Table 1:** Patient characteristics (n = 12)

Characteristics	Patients (%)
Gender	
Male	7 (58%)
Age	
Mean	82.5 (±6.4)
65‐74	3 (25%)
75‐84	2 (17%)
≥85	7 (58%)
Marital Status	
Married	7 (58%)
Other	5 (42%)
Ethnicity	
Caucasian	11 (92%)
Live Alone	
No	8 (67%)
Type of Home	
Single/Family home	5 (42%)
Apartment	7 (58%)

**Table 2:** Patient‐reported chronic conditions

Chronic conditions	Patients (%)
Mean	5 (±2.2)
Hypertension	9 (75%)
Hyperlipidaemia	6 (50%)
Asthma	1 (1%)
Diabetes	4 (33%)
Stroke	1 (1%)
COPD	1 (1%)
Renal disease	5 (42%)
Cancer	1 (1%)
Anxiety/depression	1 (1%)
Arthritis	8 (67%)
Osteoporosis	5 (42%)
Mental/cognitive illness	3 (25%)
Other	4 (33%)

Abbreviation: COPD, chronic obstructive pulmonary disease.

**Table 3:** Family caregiver characteristics (n = 7)

Characteristics	Caregivers (%)
Gender	
Female	5 (71%)
Age	
Mean	67.0 (±13.7)
<65	3 (43%)
65‐74	2 (29%)
75‐84	0 (0%)
≥85	2 (29%)
Relationship with Patients	
Spouse	5 (71%)
Child	2 (28%)

The findings are organized into three categories: understanding of illness; illness understanding and experiences of uncertainty; and influence of uncertainty on end‐of‐life discussions.

### 3.2 Understanding of illness

Participants’ understanding of illness was shaped by two main phenomena: (i) illness‐related experiences and (ii) adaptation to illness‐related challenges.

#### 3.2.1 Illness‐related experiences

Over the course of illness, patients accumulated a multitude of illness‐related experiences. Patients summarized their understanding of illness in terms of their diagnoses and procedures, previous hospitalizations and by specialists they had. Nearly all patients were aware of the symptoms they experienced on a regular basis including fatigue, breathlessness, tightness of chest and difficulty walking. Patients and caregivers also had in‐depth understanding of the self‐care behaviours of managing heart failure (e.g. monitoring their weight, adhering to their medication regimen and following dietary restrictions). Through accumulating illness‐related experiences, patients gained detailed knowledge of heart failure management. When they experienced exacerbations of heart failure, patients and caregivers were aware of the physiological changes in the body and learned when to seek medical attention. They also understood the ways in which managing heart failure interfered with their quality of life. The following quote is an example of the participant's knowledge of the self‐care behaviours of heart failure management while simultaneously sharing the ways in which following the self‐care behaviours interferes with the participant's quality of life.

"Because of the restrictions, I don't have a bowl of soup. We love onion soup at a select restaurant. I can't have that anymore. I mean I’ll steal a few tablespoons of his. But I can’t…Because it’s not only salt but it’s liquid… I’m restricted in my liquids too." Patient 12, 87 years old, female, 6 chronic conditions

#### 3.2.2 Adapting to challenges

Embedded in their experience of illness were challenges that interfered with day‐to‐day activities such as cooking, gardening and spending time with friends and family due to symptoms. Despite facing challenges regularly, participants described ways of navigating around their issues such that adapting to challenges became engrained in their lives. The following quote illustrates the ways in which the participant has reorganized their living situation in response to the challenges of illness.

"I find that when I come up the stairs, I’m a bit puffy these days. Not going down. It's just coming up. And so I think, well, they’ve put the TV in here for me so I can at least have a TV in here. And I’ve got my computer in the kitchen so for paying things. So I don’t… As I say, my office is in the kitchen, my TV’s in the dining room now. So I don't have to go downstairs." Patient 10, 88 years old, female, 4 chronic conditions

#### 3.2.3 Understanding of illness versus understanding of illness management

Although most participants were aware of their heart failure diagnosis, they did not articulate the seriousness of heart failure or the severity of their illness. Participants understood the various self‐care activities of heart failure management; however, they did not convey a sense of seriousness during discussions of the illness situation nor did they appear to grasp the severity of illness despite having advanced heart failure. Some participants understood heart failure as acute events such that ‘they had heart failure’ (Patient 12, 87 years old, female, 6 chronic conditions) during a previous hospitalization. In a few cases, patients were unaware of their diagnosis and believed that their ‘heart is in good shape’ (Patient 3, 89 years old, male, 7 chronic conditions). In one minor case, a 90‐year‐old patient with advanced heart failure and 4 other chronic conditions was adamant that she did not have heart failure but rather that she had a strong heart. The same patient became breathlessness during the interview and explained that attending appointments usually ‘wipes her out’.

Patient 7: Everything is…I’m managing fine, other than the fact that I have cancer and I have a heart…what looks like heart… I have a rapid heartbeat, you know, irregular heartbeat. I’ve forgotten what it’s called…

Caregiver 7: And chronic failure as well, right?

Patient 7: No, I don't have chronic failure of my heart. My heart's good…my heart's been pretty strong.

Many patients had experienced multiple hospitalizations and discharges from heart failure. Based on these events, participants did not appear to view their illness situations or heart failure‐related events as worrisome. Acute decompensations were not thought to be serious events compared to other cardiac events such as heart attacks and cardiac arrests. Rather, hospitalizations were thought of as a regular part of heart failure management.

"So when there's too much fluid builds up, they feel that the best way to deal with it is to set him up in the hospital and do it, you know, under supervision… It wasn’t a situation where it was like a life and death concern…it’s not like it was a cardiac arrest or something." Caregiver 6, 63 years old, wife of Patient 6 who is 76 years old and has 6 chronic conditions

### 3.3 Illness understanding and experiences of uncertainty

In spite of participants’ knowledge of heart failure management, participants still experienced uncertainty. Even though patients understood the symptoms of heart failure, they still questioned whether their experiences of symptoms were related to heart failure. For example, patients sometimes associated their symptoms to ageing or other common phenomena such as the flu. Caregivers, however, appeared more adamant that symptoms occurred as a result of heart failure or shared a different understanding of the illness event. The next exchange occurred between an 89‐year‐old patient with 4 chronic conditions and her daughter.

Patient 10: It’s just shortness of breath. Okay, so my lungs aren’t being cleared out good enough. This is probably why I have a cough all the time…my heart’s not clearing out my lungs completely…

Caregiver 10: It has happened a number of times…Sometimes it warrants an emerg visit, sometimes it clears up in a couple of days. It probably has happened at least half a dozen times.

Patient 10: It feels like the flu though.

Caregiver 10: Yeah, it feels flu‐like because she feels kind of like she doesn’t want to eat. But she’s short of… It’s always the shortness of breath that accompanies it. Which always makes me figure it’s her heart.

Patient 10: If I’ve got an upset stomach and I haven't got…I’m not short of breath, I know it's the flu. Right?

Participants did not connect the manifestations of illness or the accompanying deteriorations in health to be part of the progressive nature of heart failure. While patients and caregivers noticed fluctuations in illness and the increasing vulnerabilities in their loved ones (e.g. frequently falling and weakness), participants normalized these exacerbations of illness and did not recognize them as part of the decline towards the end‐of‐life.

"Every month there was a new crisis. But they weren’t all heart‐related. Because there was the fall. There was the heart thing and the fall in the autumn. Then there was the actual when you fell. Then there was the Christmas ornament cut. Then there was something else in March. And then the hospitalization in May. So it was sort of like…every month was a new adventure. And they all took their toll, right. Even the ones that weren’t directly related to her heart, they still took a toll on her… But now it’s sort of normal." Caregiver 10, 55 years old, daughter of Patient 10 who is 89 years old with 4 chronic conditions

### 3.4 Influence of uncertainty on end‐of‐life communication

In addition to the absence of connection between manifestations of illness and the progressive declining nature of heart failure, most participants had not engaged in prior end‐of‐life discussions with family members or health‐care providers. Even among participants who shared their awareness of death as an inherent part of their future, they had not engaged in end‐of‐life discussions with their family members or clinicians. Rather, participants were focused on maintaining their current level of health and being able to manage their illness. Several participants shared their hope to maintain their current level of health for years to come. Hence, the common approach that patients and caregivers appeared to take was to wait until further decline to engage in end‐of‐life discussions.

Interviewer: So do you think it's important for all of your healthcare providers to know about your preferences?

Patient 8: To be honest with you, it's something I put into the future… And that’s probably wrong. The future is probably now…I haven’t had any conversations, no, about this. (87 years old, female, 6 chronic conditions)

‐‐‐‐

"But you know, he’s not there yet. He’s still in pretty good shape…from my experience, and I could be wrong, but I know when the patient is sort of deteriorated to the point where you know there's no return. So I think my husband is in pretty good shape." Caregiver 6, wife of Patient 6 who is 76 years old with 6 chronic conditions

In one rare case, a patient and caregiver dyad spoke frankly and openly about their end‐of‐life preferences and was the only participant pair that had prior end‐of‐life discussions with their clinicians. The patient and caregiver were aware of their illness situation and were knowledgeable of the self‐care behaviours of heart failure management. Despite acknowledging the uncertainty that surrounds how decline in illness would transpire, the patient and caregiver had discussed their preferences for care which included being cared for in their home for as long as possible and not wanting technological interventions at the end‐of‐life.

Interviewer: …if your health condition were to suddenly decline, what would be most important to you guys?

Caregiver 1: I would look after him as long as I could. If I needed help, then I would have to get it…we both don’t want to be kept alive on machines. We both have that so we know what is important to us…I think we gave [cardiologist] a copy of a DNR, and we’ve done the power of attorneys for medical care…I think they know our concerns, and as I say, we’ve given them forms of, you know if it comes down to it, we don’t want to be kept alive artificially. (86 years old, female, wife of Patient 1)

Patient 1: [My wife] and I both, are quite prepared that at sometime we are going to die, and try to organize our lives to make it reasonably simple for the other partner and for the kids. (86 years old, male, 3 chronic conditions)

## 4 DISCUSSION

This study explored what older adults with advanced heart failure and family caregivers understand about their illness and how it relates to experiences of uncertainty and perceptions of end‐of‐life discussions. Participants’ understanding of illness was shaped by illness‐related experiences and the ability to adapt to the challenges of illness. Adapting to challenges occurred regularly such that making modifications to living situations had become a normalized process. One example of this process of adaptation is the perception that hospitalizations are a routine part of heart failure management. This raises a question as to whether the cumulative experience of being discharged from hospitalizations in heart failure distorts one's perception of illness such that patients and caregivers gain a false sense of recovery despite the progressive nature of illness and the threat of mortality. This may be a phenomenon that is unique to people who experience intermittent decline. Our findings corroborate prior studies^27^, ^28^, ^29^; in a study of older adults with advanced heart failure, Klindworth and colleagues found that older patients do not perceive heart failure as a life‐limiting illness.^28^ In a longitudinal study of patients with advanced chronic obstructive pulmonary disease, an advanced illness with a similar illness trajectory to heart failure, Pinnock and colleagues found that patients reported having been ‘restored to normal and the threat had receded’ following an exacerbation.^29^ Consequently, the patients with chronic obstructive pulmonary disease felt that the presence of death was not thought to be imminent and had not discussed end‐of‐life care with their providers. Clinically, acute decompensations of heart failure are understood to be part of the progression in illness towards the end‐of‐life. Our findings suggest that patients and caregivers may conceive exacerbations as temporary health states and therefore do not perceive a need for end‐of‐life discussions. Clinicians should be aware of such misperceptions as they can delay end‐of‐life discussions. When end‐of‐life discussions are not had until very late or too late in the illness trajectory, patients and caregivers may not receive care concordant with their wishes.

Interestingly, despite having knowledge of heart failure management, participants did not share an understanding of the consequences of their illness. Many patients and caregivers understood their illness situation in terms of their experiences in the health care system and with their illness (e.g. illness management routines, symptoms and hospitalizations). Similarly, Bell‐Davies found that caregivers’ understanding of heart failure included keeping track of GP and hospital visits and arrange appointments, and based on these experiences, obtain developed explanations for symptoms and illness‐related experiences of heart failure.^30^ In neither study were the consequences of illness often discussed as part of understanding heart failure. While our sample of patients and caregivers received patient education under the specialized heart failure clinic model of care and despite its effectiveness in teaching the importance of self‐care behaviours, it often does not include the consequences of illness. As an example, the Canadian Cardiovascular Society has defined the scope of patient education to cover self‐care behaviours and medication management. However, information on the consequences of heart failure or the various modes of decline and death in older adults are often absent from patient education in models of chronic disease management implemented in many western health care systems.^31^ This is a possible explanation as to why, despite having detailed knowledge of heart failure management, patients and caregivers may not have shared an understanding of the illness trajectory and the consequences of illness. This is unsurprising as models of chronic disease management in western health care systems overwhelmingly emphasize self‐management and preventions of further decline in illness.^32^, ^33^ While there are clear benefits of self‐management education, it often does not address the inevitable decline that accompanies advanced illness. As a result, addressing the consequences of illness and end‐of‐life communication are seldom integrated into chronic disease management to normalize the progression towards the end of life.

Incorporating the consequences of illness into patient education is one way to bridge the gap between chronic disease management and end‐of‐life care.^34^ By helping patients and caregivers understand the implications of their illness context, it can prompt patients and caregivers to think about what may be important at their stage of illness and life, and provide clinicians with an opportunity to align care plans to patients’ goals and values. This may be particularly important to older adults given the difference in their goals and priorities in life.^35^, ^36^ These opportunities to discuss goals and priorities can be critical for patients and their families; a seminal study reported that people consider being able to say goodbye, remembering personal accomplishments and resolving unfinished business to be important at the end‐of‐life.^37^ Current care practices that are not inclusive of opportunities for recurring end‐of‐life communication to happen mean that opportunities for closure or to identify goals and priorities may be missed. In a study of individual's awareness of dying, Stacey and colleagues found that despite the mounting information that patients and family members received from various clinicians, participants were still unaware that their loved ones were dying and could not grasp the implications of what they had been told.^38^ There is a need to better support patients and caregivers in understanding their illness situations as well as the consequences of their illness to improve the end‐of‐life experience for patients and caregivers. As well, patients and caregivers may need time to process information related to the reality of their illness situations which further underscores the need for earlier integration of end‐of‐life discussions.

### 4.1 Alignment to the reconceptualized uncertainty in illness theory

Several findings from the study align with the Reconceptualized Uncertainty in Illness Theory. It appears that the accumulation of illness‐related events (e.g. symptoms and hospitalizations) contributed to participants’ understanding of illness‐related experiences. This was most notable in participants’ ability to recognize the onset of heart failure exacerbations. Family members and informal caregivers, who provide support to patients, also appeared to reduce uncertainty as they supplemented patients’ understanding of illness. Participants’ ability to adapt to illness‐related challenges and further integrating the process of adaptation into patients’ and caregivers’ lives support the notion that individuals adapt to a new way of living upon experiences of prolonged uncertainty.

Despite the accumulation of experiences of illness‐related events and gained familiarity over time, both patients and caregivers struggled to recognize manifestations of illness as part of the progression towards the end‐of‐life. Moreover, the Reconceptualized Uncertainty in Illness Theory recognizes caregivers to be a source for understanding illness‐related events. In this study, we found that caregivers did supplement patients’ understanding of illness, and therefore, reduced uncertainty in illness. However, we also found that caregivers experience uncertainty themselves. Importantly, it appears that when patients and caregivers experience similar uncertainties in illness, both are left with gaps in their understanding. In advanced illness, the consequences of prolonged uncertainty can be detrimental as this uncertainty can contribute to delays in or a lack of end‐of‐life discussions when both patients and caregivers do not understand manifestations of heart failure as part of the progression in illness. The fact that caregivers also experience uncertainty is particularly relevant in the current health care environment as their participation in illness management is increasing in patient care. Although the Reconceptualized Uncertainty in Illness Theory was developed to focus on the patient's experience of uncertainty, given the changing landscape of health care and the increasing reliance on informal caregivers in care, it may be necessary to adapt the theory to capture the uncertainty in illness experienced by both patients and caregivers.

### 4.2 Strengths and limitations

This study explored experiences of uncertainty and end‐of‐life communication in the context of what patients and caregivers understand about their illness. This is of critical importance in studies of end‐of‐life communication, as understanding of illness sets a logical foundation for one to perceive a need to engage in end‐of‐life discussions. The findings of this study provide insights from the patient and caregiver perspective, which can be leveraged to improve end‐of‐life communication for people living with heart failure.

The findings of this study should be interpreted within its limitations. All patients in this study had multiple chronic conditions; however, given the focus on heart failure to understand its unique illness trajectory, it is possible that the complexity of multimorbidity in participants’ understanding of illness was not fully captured. There is also a question regarding the extent to which participants shared their thoughts given the sensitivity of the topic explored. Furthermore, this study did not try to discern whether or not participants were willing to articulate their understanding or truly lacked understanding of the consequences of illness. Nonetheless, the findings highlight aspects of illness that clinicians may need to proactively address in care, particularly as it can impact patients and caregivers’ end‐of‐life experience. Our sample of caregivers is also small and diverse (i.e. includes spouses and children). A greater sample would be helpful and is needed to discern the different experiences of engaging in end‐of‐life discussions. The study participants were recruited from one specialized outpatient clinic where clinicians are trained in heart failure management as well as teach patient education to their patients and caregivers. All but one participant was Caucasian, which also does not reflect the entire heart failure population. This is, in part, a reflection of the inclusion criteria that excluded patients who are not fluent in English. How these findings would hold among a culturally diverse sample of patients is unknown, as previous research has found variations in end‐of‐life preferences in different populations. All of these factors limit the transferability of the study findings.

## 5 CONCLUSION

This study explored older adults with advanced heart failure and family caregivers’ understanding of illness and their experiences of uncertainty and end‐of‐life discussions. Patients and caregivers may have knowledge of heart failure management, and yet, there may be gaps in patients' and caregivers’ understanding of the consequences of illness. Clinicians should be aware of what older adults and caregivers understand about the consequences of their illness to ensure that their care plans align with their preferences and needs, as they age and advance in their illness towards the end‐of‐life. Importantly, this study highlights that both patients and caregivers experience uncertainty in illness, which currently remains unaccounted for in the Reconceptualized Uncertainty in Illness Theory. Future studies are needed to test whether addressing the consequences of illness in heart failure management can improve understanding of illness and earlier integration of end‐of‐life communication for older adults and their family caregivers.

## Data Availability

Data cannot be shared publicly because of restrictions found in the research ethics approval. Study participants did not provide consent to have transcripts of their interviews to be shared publicly. Deidentified data for researchers who meet the criteria for access to confidential data may be requested from the Mount Sinai Hospital Research Ethics Board. For more information, please contact Jennifer.im@mail.utoronto.ca.
